# Self-reported health outcomes and medical complications at 6- and 8-year follow-up after direct skeletal fixation in individuals with bilateral transfemoral amputations

**DOI:** 10.1097/MRR.0000000000000677

**Published:** 2025-07-11

**Authors:** Diana Toderita, Charles Handford, Arul Ramasamy, Paul Hindle, Jonathan Kendrew, Anthony M.J. Bull, Louise McMenemy

**Affiliations:** a Department of Bioengineering, Centre for Injury Studies, Imperial College London, London; b Academic Department of Military Trauma and Orthopaedics; c Queen Elizabeth Hospital Birmingham, Royal Centre for Defence Medicine, Birmingham, UK; d St Paul’s Hospital, University of British Columbia, Vancouver, British Columbia, Canada

**Keywords:** amputation, direct skeletal fixation, osseointegration, transfemoral, trauma, veterans

## Abstract

Direct skeletal fixation (DSF) involves attaching the intramedullary portion of a prosthesis directly to the skeletal residuum, providing an alternative for amputees unable to mobilise with socket-based prostheses. This study investigates the effects of DSF on physical and mental health at 6- and 8-year follow-up for military bilateral transfemoral amputees in the UK. Eight male bilateral transfemoral military amputees who underwent implantation with the Osseointegration Group of Australia–Osseointegration Prosthetic Limb prosthesis consented to participate in the study. All patients are routinely reviewed annually in a dedicated clinic, and this paper reports the 6- and 8-year follow-ups. Patient-reported outcomes were assessed using the Short Form Health Survey (SF-36). Complications data were recorded at the 8-year follow-up. The SF-36 physical component score significantly increased from preoperative levels at 6 years (median: 29 vs. 47; *P* = 0.003) and 8 years (median: 29 vs. 45; *P* = 0.024). The SF-36 mental health component score improved significantly at 6 years from preop (median: 39 vs. 57; *P* = 0.011). Among 16 femoral residuums, there was one explantation because of infection at 8.5 years postimplantation, and two cases were managed with long-term suppressive antibiotics. A total of 17 additional procedures were performed on nine residuums: 11 for soft tissue revision, five for infection, and one for fracture repair. This research adds to the growing evidence base that DSF has the potential to enhance the health and well-being of amputee veterans and potentially the broader amputee population. Medical complications remain an important consideration.

## Introduction

Between 2003 and 2014, military operations in Afghanistan resulted in 265 UK military amputees [1]. Advanced rehabilitation and modern prostheses provided by the Defence Medical Services facilitated a return to an unprecedented level of physical activity; however, some amputees faced significant challenges mobilizing with traditional socket-suspended prostheses because of soft tissue complications, pain, or discomfort [2]. This was particularly true for blast injury patients who may develop heterotopic ossification [3]; this condition continuously alters the residuum shape necessitating repeated socket fitting. In addition, delicate skin grafts on the residuums can break down under traditional sockets, reducing mobility [4]. These issues can lead to prosthetic abandonment and wheelchair dependence [2,5,6].

For these individuals, direct skeletal fixation (DSF) offers a promising alternative fixation method to traditional socket-based prosthetics. This method involves the insertion of a percutaneous titanium implant into the residual bone [7,8]. It allows for the direct loading of the bone through the implant, protects the soft tissue of the residual limb, and enhances proprioception [9]. By eliminating the need for socket-based technology, this approach avoids the complications and limitations associated with socket use.

Studies have reported short and medium-term outcomes of DSF up to 5 years postoperation, showing improvements in prosthetic use [8,10–13], walking ability [14], and overall quality of life [10,15]. The only studies presenting data beyond 5 years show a high reliance on assistive devices after 7 years in a heterogenous group [16]. In a group of 11 patients with 15 years’ follow-up, there were no significant differences in prosthetic use and mobility compared with preoperative scores [17].

Research on the use of DSF in military blast injury patients globally at medium-term follow-up reveals a significant knowledge gap. This is particularly regarding infection-related complications in blast-related traumatic amputation residuums that may arise from the contamination of bone and soft tissue at the time of injury. Military amputees may experience different functional outcomes than civilian amputees, as they are generally younger, highly motivated, and eager to return to high-intensity activities. The UK military rehabilitation programme, known for its high intensity and focus on achieving high levels of functionality, likely exceeds the goals of civilian rehabilitation programs, such as those proposed by the British Association of Chartered Physiotherapists in Amputee Rehabilitation [18] and the Dutch evidence-based guidelines for amputation and prosthetics of the lower extremity [19], which primarily aim to restore competence in activities of daily living. To our knowledge, no previous DSF studies have exclusively focused on a prospectively collected group of military amputees, highlighting the need for targeted research in this population.

Most research has focused on unilateral amputees, with limited knowledge about the outcomes for bilateral amputees. In the UK, 26 military personnel sustained bilateral transfemoral amputations between 2003 and 2014 [20]. Of these, eight underwent DSF between 2014 and 2016. The first seven UK military veterans with bilateral transfemoral amputations who underwent DSF surgery were reviewed and the published data demonstrated improved quality of life at 2 years postsurgery, with minimal complications [21]; however, beyond 2 years, physical and mental health outcomes for bilateral transfemoral military amputees with DSF are not well described. A combined patient-reported and medical complications study would provide a comprehensive understanding of this DSF cohort.

This study aimed to determine the effects of DSF surgery on physical and mental health outcomes at 6- and 8-year follow-up for the first cohort of bilateral transfemoral UK military amputees who reported socket-related issues resulting in selection for DSF.

## Methods

This prospective study analyzed the outcomes of DSF surgery for eight male military veterans with bilateral transfemoral amputations, all of whom were injured by blast. All military patients who underwent DSF surgery between 2014 and 2016 were included in the study, seven of whom were previously included in the 2-year report [21]. The median age at the time of DSF surgery was 27 years [interquartile range (IQR): 24–30 years], and the median time between initial amputation and DSF surgery was 68 months (IQR: 56–69 months). The delay between injury and DSF was because of failure to achieve adequate mobility with socket-suspended prostheses, which formed part of the inclusion criteria to receive DSF. Individuals were initially fitted with socket-based prostheses but transitioned to DSF at the end of their prosthetic pathway because of limited functional mobility, spending at least 50% of their ambulatory time using a wheelchair. Participants had undergone bilateral implantation with the Osseointegration Group of Australia–Osseointegration Prosthetic Limb (OGAP-OPL) prosthesis. Except for one case, all surgeries were performed as single-stage procedures following the Osseointegration Group of Australia Accelerated (OGAAP) Protocol-2 [22]. Comprehensive rehabilitation was conducted at the Defence Medical Rehabilitation Centre UK following both the initial amputation surgery and subsequent DSF surgery.

Patient-reported outcomes were evaluated with the Short Form Health Survey (SF-36), from which scores for physical health component (PCS) and mental health component (MCS) were derived [23]. These evaluations were conducted before surgery and at 6 and 8 years after surgery. The SF-36 was administered in person preoperatively and at the 6-year follow-up. At the 8-year follow-up, five out of six participants completed the SF-36 in person during scheduled visits, while one participant completed it remotely via a secure online platform. The median follow-up was 70 months (IQR: 63–78 months) at 6 years, and 100 months (IQR: 95–102 months) at 8 years. Complications data were collected, including infection, soft tissue complications, need for stem explantation, and any other issues with the extra medullary prosthesis. Before surgery, participants consented both to undergo the procedure and to the use of their routinely collected complications and SF-36 data for research purposes. Accordingly, the complications, preoperative, and 6-year questionnaire data were collected during routine in-clinic visits under the original consent and subsequently obtained from participants’ medical records. All participants provided written informed consent under the Imperial College Research Ethics Committee approval (ICREC 22IC7601) at their 8-year follow-up visit, when they completed the SF-36 Questionnaire.

Statistical analysis was performed using IBM SPSS version 28. Scores from the three time points (preoperative, 6- and 8-year follow-up) were compared using one-way repeated measures analysis of variance, followed by pairwise comparisons with Bonferroni corrections. Participants acted as their own controls.

## Results

### Self-reported health outcomes

**Fig. 1:**
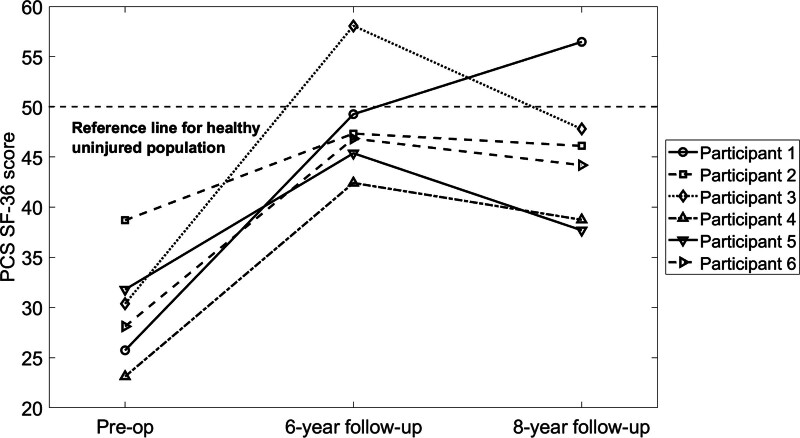
Individual trajectories of the PCS of the SF-36 Questionnaire for transfemoral amputees with bilateral DSF. DSF, direct skeletal fixation; PCS, physical health component score; SF-36, Short Form Health Survey.

**Fig. 2:**
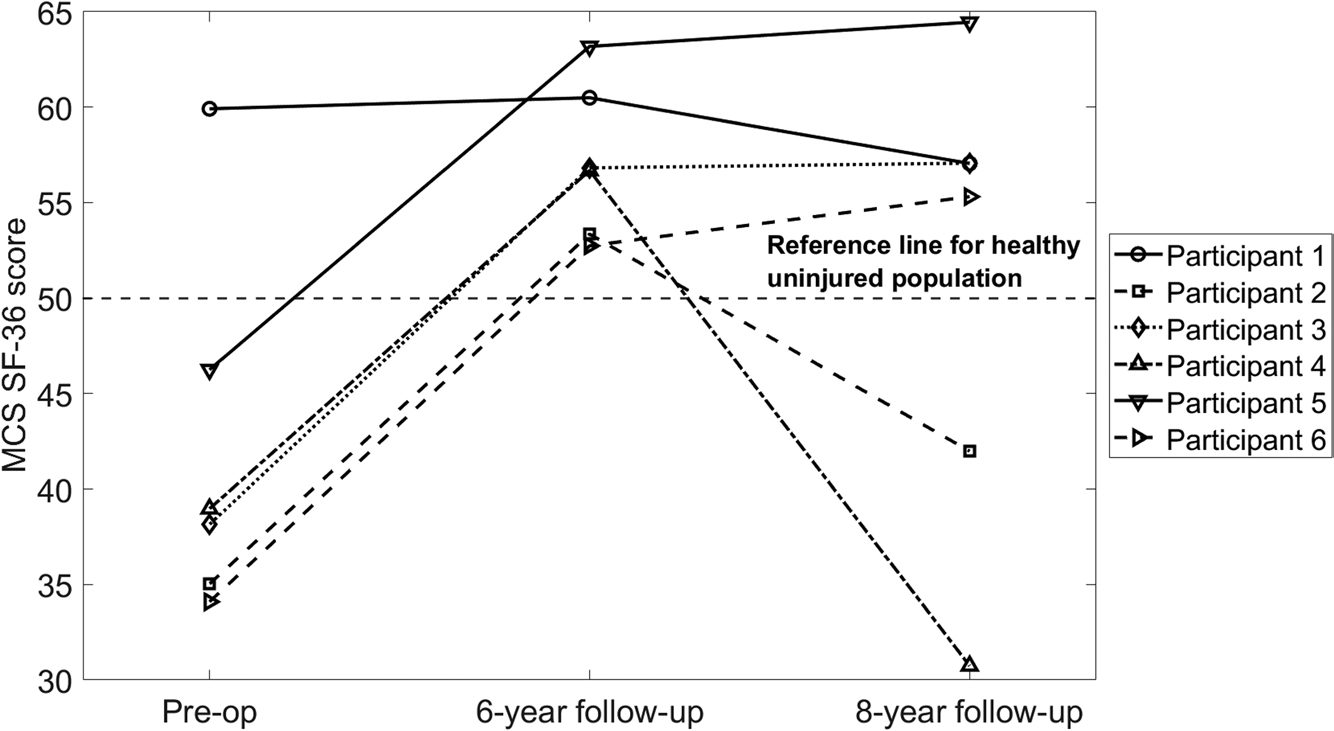
Individual trajectories of the MCS of the SF-36 Questionnaire for transfemoral amputees with bilateral DSF. DSF, direct skeletal fixation; MCS, mental health component score; SF-36, Short Form Health Survey.

Full follow-up data for the SF-36 were available for six of the participants (Figs. 1 and 2). A median score of 50 represents PCS and MCS scores of an uninjured population [23]. The SF-36 PCS improved significantly by the 6-year follow-up, increasing from a preoperative median of 29 (IQR: 24–35) to 47 (IQR: 43–54) (*P* = 0.003). At the 8-year follow-up, the SF-36 PCS median was 45 (IQR: 38–52), a significant increase from the preoperative median of 29 (IQR: 24–35) (*P* = 0.024). There was no significant difference between the 6- and 8-year follow-up scores. No participants exceeded the normative SF-36 PCS threshold of 50 points preoperatively, one did at 6 years, and one at 8 years. There was a statistically significant increase in the SF-36 MCS at the 6-year follow-up (median: 57, IQR: 53–61) compared with preoperative scores (median: 39, IQR: 32–52) (*P* = 0.011). At 8 years, the median MCS was 56 (IQR: 38–64), which did not differ significantly from preoperative values. One participant exceeded the normative SF-36 MCS preoperatively, six at 6 years, and four at 8 years.

### Complications

A total of eight patients, 16 femoral residuums, have had transfemoral DSF for over 8 years, with a median of 9 years (8–10 years). Two patients (12.5%, 2/16) were placed on long-term suppressive antibiotics for single residuum infection at a time of 4.5 and 6.8 years postinsertion. One of these cases resulted in explantation at 8.5 years postimplantation (6.25%, 1/16), following 21 months of suppression. The other case at the time of writing has been on suppression for 44 months with no recurrent symptoms of infection and good function. Within this cohort there has been one (6.25%, 1/16) fracture 5 months after the index surgery fixed with a dynamic hip screw following trauma with a subsequent return to normal activities; this was reported in the initial 2-year follow-up paper [21].

Nine (56%, 9/16) residuums have undergone further procedures, with a total of 17 procedures. Four residuums underwent one procedure, three residuums two procedures, one residuum three procedures, and one residuum four procedures. Eleven (65% 11/17) procedures were for soft tissue revision in a total of eight (50%, 8/16) residuums, five (30% 5/17) were for infection on three different residuums, and one (5% 1/17) procedure was for fracture. In regard to infection, three of the five procedures were done on one residuum culminating in explantation. Of the remaining two procedures, one was for a stitch abscess in the soft tissues and the other for infection, including the bone, after which the patient was placed on long-term suppressive antibiotics. There are two (25%, 2/8) bilateral amputees who at the time of writing, have not had to undergo any additional surgery who are at 9 and 8 years postimplantation.

The median time to first soft tissue revision was 26 months (*n* = 8, 11–84 months). The median time to first surgery for infection was 49 months (*n* = 3, 22–61 months). The case operated on at 22 months was for a stitch abscess with complete resolution poststitch removal.

## Discussion

This study presents the 6- and 8-year follow-up outcomes for the first cohort of UK military personnel who underwent DSF surgery and provides critical insights into the medium-term physical and mental health implications of this procedure. Building upon the initial findings at the 2-year mark, which reported significant improvements in physical abilities and quality of life with minimal complications [21], the extended follow-up data offer a comprehensive understanding of the effects of DSF in this unique population.

The SF-36 Questionnaire captured data on limitations in activities of daily living and explored how these limitations impacted overall quality of life. Participants demonstrated significant improvements in the physical health component score after 6 years, reaching levels close to those of uninjured individuals. The physical health benefits observed at the 6-year follow-up reaffirm the initial positive outcomes reported at 2 years [21]. Similarity in outcomes at 2 and 5 years postsurgery has been reported for persons with unilateral implantation with the Osseointegrated Prosthesis for the Rehabilitation for Amputees (OPRA) system [10]. This suggests that the immediate benefits of DSF not only persist but also stabilize over an extended period, underscoring the procedure’s potential as a solution for amputees who are not able to tolerate traditional prosthetic sockets.

Despite a slight decrease in physical health at the 8-year follow-up for some participants, their scores remained significantly higher than preoperative levels and approximated that of an uninjured population. Of course, these are patients with polytrauma, and therefore, the decline may be because of other injuries as opposed to being directly related to DSF. This finding is consistent with previous reports at 7 and 10 years postoperation for unilateral transfemoral amputees treated with the OPRA system [17]. The decline observed in this study may be attributed to the natural aging process, which may lead to reduced muscular reserve and overall physical capacity [24]. For amputees, this reserve may be much smaller than that of able-bodied individuals because of the absence of joints and muscles, placing greater strain on the residual muscles [25] and making them more susceptible to declines in physical health [26–29]. In addition, multiple amputations can further decrease quality of life and physical function.

The follow-up data indicate significant improvements in the mental health component score at the 6-year mark compared with preoperative levels, aligning with findings at 2 years [21]; however, by the 8-year follow-up, this significant difference had disappeared. While some participants maintained consistent mental health scores, others experienced declines. This variability underscores the personalized nature of outcomes among individuals with limb loss, influenced by a range of factors including anatomical variability, personal experiences, and individual circumstances. Mental health outcomes over time may be influenced by elements such as the psychological distance from conflict zones and military service, leading veterans to experience varying challenges as they transition to civilian life [30], unrelated to the DSF surgery. Physical combat injuries among UK armed forces personnel deployed to Afghanistan have been linked to poor mental health outcomes [31]; however, those who sustained amputations had notably lower odds of reporting mental health issues compared to those with nonamputation injuries, and showed no significant differences compared with noninjured personnel [31,32]. The observed decline in mental health at 8 years in this study may also correlate with increasing age and possible physical health declines for some participants, whereas others maintained high mental health scores despite lower physical health scores, highlighting the intricate relationship between physical and mental well-being. It is worth emphasizing that this relationship was consistent through to the 6-year follow-up, with all participants showing improvements in both physical and mental health component scores relative to their preoperative baseline scores. By the 8-year mark, however, trajectories diverged, reflecting increased variability in long-term outcomes.

Our results indicate an implant survival rate of 93.75% (15/16 residua) at 8 years. Because of the heterogeneity, short length of follow-up, and multiple available devices, the long-term survivorship is still uncertain. A concern in this veteran cohort was that they may experience higher deep infection and subsequent explantation rates because of the polymicrobial, including fungal, contamination associated with military blast injury; however, this does not appear to be the case, and our survivorship appears comparable to published data. Outcomes using the OPRA Implant System showed a survival rate of 92% (post-OPRA formalized protocol) or 60% (pre-OPRA formalized protocol) at 11 years, with an overall implant removal rate of 28% (5/18), all of which were ultimately shown to be infected [10]. Furthermore, in that group, they report two individuals on long-term suppressive antibiotics, which would give a rate of 15%, and this is higher than our rate of 6.6% (1/15) if explanted cases are excluded. A further study reported a 10-year osteomyelitis risk of 20% and a 10-year explantation risk of 9% using the OPRA Implant System [33]. There are no published outcomes beyond 8 years for the OGAAP-OPL, but the published literature reports low implant failure rates for infection [34].

Peri-implant fractures are a reality of orthopedic implants; the fracture rate in this cohort is low at 6.25% (1/16), and we would argue is a marker of increased functionality. We found that in this case the fracture was managed successfully with dynamic hip screw fixation and a good return to activity. This is supportive of the good outcomes following peri-implant fracture around transfemoral DSF presented in a large retrospective study that found a similar fracture prevalence [35]. Fractures in our bilateral DSF cohort are a reflection of their increased activity levels and return to normal activities.

Soft tissue management is a challenge in all amputees and often a reason for conventional socket abandonment. In DSF soft tissue revisions for irritation, debulking and stabilization are common, but reporting and classification can be variable; with modern techniques the literature suggests a rate of soft tissue revision surgery at 16–36% [12,22,36]; however, it must be noted that these studies have shorter follow-up periods and given the nature of soft tissue injury associated with blast injury, this specific cohort may be more at risk of problematic soft tissues, which may explain our rate of 50% (8/16) of residual limbs requiring at least one soft tissue revision. In addition, these patients represent the first to undergo DSF at this specific center and as such there may be a learning curve in regard to the amount of debulking required and the nuances associated with forming the soft tissue residuum and stoma; those who have undergone DSF more recently may, therefore, have a different outcome. This will be assessed through further data collection.

When evaluating outcomes, it is important to acknowledge that although DSF offers improved results by eliminating socket-related issues, rehabilitation plays a crucial role in achieving optimal outcomes by focusing on neuromusculoskeletal capacity improvement [37]. This study’s participants underwent comprehensive and intensive rehabilitation care at the Defence Medical Rehabilitation Centre UK, where they trained with the new prosthesis and adopted new compensatory movement strategies to enhance musculoskeletal function and overall health. Therefore, translation to other health services involves the need for appropriate intensive rehabilitation to hope to recreate similar results. In addition, translation of results to civilian populations should also be undertaken with caution. Military populations are preconditioned with physical activity and intensive training, which may improve physical and mental scores when compared with civilian counterparts. Reflecting this, the National Institute for Health and Care Excellence has now published evidence-based recommendations for the civilian population in the UK on DSF of limb prostheses.

A limitation in this study is the small number of participants, which restricts the generalisability of these findings to a broader amputee population. Inclusion of mobility data would strengthen the results, but because of limited longitudinal follow-up (available in only two participants at all three assessment points), this was unfortunately not possible for this cohort. Mobility data were missing when participants completed the SF-36 Questionnaire online or during in-person visits, because of limited appointment time and occasional lack of dedicated space to conduct the mobility tests. Larger trials and longer follow-up periods extending beyond 8 years are necessary to fully evaluate the clinical implications of the DSF procedure.

### Conclusion

This study highlights that DSF offers a viable option for bilateral transfemoral amputees unable to mobilize effectively with traditional socket-suspended prostheses. DSF has the potential to provide long-term physical and mental health benefits and allow veterans to be fully integrated in society. Nevertheless, there is a risk profile associated with the intervention, and it must be viewed as a second-line intervention following failure of conventional socket prosthetics. Continued long-term follow-up of all who undergo DSF is essential to further understand the effect of this procedure on quality of life beyond 8 years. A specific registry would help facilitate this.

## Acknowledgements

The authors would like to thank the Birmingham Osseointegration Group (Mr. Demetrius Evriviades, Mr. Paul Fenton, Miss Deborah Foong, and Mr. Michael Parry) for their valuable collaboration throughout this work.

This report has been produced with funding from the Office for Veterans’ Affairs in April 2023 as part of an independent research initiative. The views, findings, and conclusions expressed herein are those of the authors and do not reflect the policies or positions of the UK Government.

Conceptualization: L.M., A.R., A.M.J.B., D.T., C.H. Funding acquisition: L.M., P.H., A.R., A.M.J.B. Data acquisition: D.T., C.H. Formal analysis: D.T. and C.H. Data interpretation: D.T., C.H., A.R., P.H., J.K., L.M., A.M.J.B. All authors read and approved the final manuscript.

### Conflicts of interest

There are no conflicts of interest.
